# Coupled Multiphysics Numerical Simulation of a Thermo-Elastohydrodynamic O-Ring in a High-Pressure Hydrogen Gas Quick Coupler

**DOI:** 10.3390/polym17111478

**Published:** 2025-05-26

**Authors:** Artur Wodołażski

**Affiliations:** Department of Energy Saving and Air Protection, Central Mining Institute, Plac Gwarkow 1, 40-166 Katowice, Poland; awodolazski@gig.eu

**Keywords:** FSI—fluid–structure interaction, O-ring seals, high-pressure hydrogen, moving mesh, quick connector

## Abstract

In this study, a novel mechanical fluid–structure interaction (FSI) model is developed to analyze and discuss high-pressure hydrogen flow in a quick coupler under various operating conditions. The transient-state behavior is investigated with respect to different temperatures, hydrogen pressures, and O-ring thicknesses, which directly affect the compression and deformation of the seal. High-pressure hydrogen flow, which may lead to seal damage or failure, is of growing concern due to the increasing use of hydrogen in refueling stations, a sector expected to play a key role in the future of clean energy infrastructure. This study aims to introduce a coupled multiphysics approach by integrating the Finite Element Method (FEM) for solid mechanics with the Finite Volume Method (FVM) for hydrogen flow modeling. The coupling model shows nonlinear interactions between flowing hydrogen and the deformable polymer seal. The results of this work are expected to enhance both the design and performance of high-pressure hydrogen quick couplers, especially for applications in next-generation hydrogen refueling stations, where durability, sealing efficiency and safety are critical.

## 1. Introduction

In the face of the growing energy crisis and growing environmental problems related to greenhouse gas emissions from the combustion of fossil fuels, more attention is being paid to alternative sources of energy with low carbon emissions. In the context of global decarbonization goals, hydrogen is emerging as one of the key links in the future energy mix due to its renewable origin, high energy efficiency and climate neutrality [1]. Considered as one of the most promising fuels of the 21st century, hydrogen can play a significant role in counteracting environmental degradation and mitigating the effects of the depletion of traditional energy resources [2,3]. However, the characteristic physicochemical properties of hydrogen, such as very low density, high specific energy, and wide flammability range, make it a highly flammable and ignition-prone substance. As a result, safety issues, especially in the context of fires and explosions, constitute a serious limitation for its wide application [4]. The infrastructure of hydrogen refueling stations relies on high-pressure quick connectors, which ensure the safe and reliable connection of gas transmission systems under demanding conditions. Their performance is critical throughout the hydrogen life cycle, from production and transport to storage and end-use in vehicles. Consequently, the development of advanced connection technologies that are capable of withstanding extreme pressures and temperatures remains a key research focus, supporting both the safety and efficiency of hydrogen refueling stations (HRSs). In this context, quick-connector seals play a vital role in maintaining system integrity, making them essential components in industrial applications requiring secure and leak-tight connections. Their basic function is to ensure tightness by preventing leaks of working media and limiting the penetration of contaminants from the environment into the system, especially in the contact area between the seal and the mating surface. In the context of hydrogen quick-release seals used in hydrogen refueling stations at pressures of 15–70 MPa, the behavior of the seal under extreme load conditions is crucial for the process safety. High contact stresses and heat generated due to friction lead to the local overheating of the seal material, which may result in its premature wear or failure [5]. The analysis of these phenomena requires the use of advanced computational methods, such as the nonlinear Finite Element Method (FEM), which takes into account both geometric and material nonlinearities, as well as large deformations in the deformed contact zone. FEM models allow for the representation of actual seal operating conditions, including, among others, shaft misalignment, load deflections, and the influence of compression springs. Particular attention should be paid to the thermomechanical behavior of the seal–coupling interface because high temperatures can significantly change the stiffness of the sealing material and reduce its resistance to compression [6,7,8]. Contact analysis also takes into account the contact force, the distribution of shear stresses, and the development of micro-strains at the contact interface. Due to the strongly nonlinear resistance torque characteristics in quick connectors during hydrogen flow, they are also used as regulating elements with a simultaneous leak protection function. Their mechanical resistance, dynamic tightness, and ability to operate under cyclic pressure and temperature loading conditions determine their suitability for applications in hydrogen infrastructure with increased safety requirements, such as industrial H_2_ distribution systems and refueling stations [9]. In the design of high-pressure quick connectors used in hydrogen refueling systems, O-ring seals play a key role; they must be made of highly specialized materials that are resistant to hydrogen and extreme pressure and temperature conditions. Traditional elastomers, such as NBR (nitrile rubber) or EPDM (ethylene–propylene rubber), exhibit too high permeability to hydrogen and are susceptible to chemical degradation, which is why they do not meet the safety requirements in hydrogen applications [10,11]. The most commonly used sealing materials in quick connectors include perfluoroelastomers (e.g., Kalrez), characterized by exceptional chemical resistance and very low permeability to hydrogen, which make them one of the most reliable solutions for this type of application; fluoroelastomers (FKM, e.g., Viton), which exhibit good resistance to chemicals, but their durability when in long-term contact with hydrogen may be limited, especially in dynamic environments; HNBR (hydrogenated nitrile butadiene rubber) is used in applications requiring moderate hydrogen resistance and good mechanical stability, while PTFE (polytetrafluoroethylene, commonly known as Teflon) offers excellent chemical and thermal resistance and is often employed in the form of composite seals for extreme operating condition. O-rings play a key role in hydrogen quick connectors, ensuring tightness during both the connection and disconnection of the connector under high pressure. Their effectiveness directly affects the minimization of the risk of hydrogen leakage, which, as an odorless, light, easily flammable and difficult to detect gas, poses a significant safety risk [12,13,14]. The effect of hydrogen temperature and pressure on seals made of perfluoropolymers, as well as the flow rate, has a significant effect on the degree of O-ring compression. These issues are crucial from the point of view of safety and minimizing the risk of leakage at hydrogen refueling stations, which will play an increasingly important role in connection with the growing importance of alternative fuels. Most research works are focused on the analysis of the steady-state flow field under different valve opening conditions. CFD models can overcome these limitations, provide deeper insight into the fundamental physical phenomena, and simulate the combined effects of parameters on heat and mass transfer during the opening–closing of the quick connector in order to perform high-performance and silty operations during the refueling/use process of the vehicle. CFD models are usually associated with high computational costs. Xu et al. [1] used numerical simulations to investigate the influence of different hydrogen leak parameters on the complex hydrogen diffusion dynamics in large rectangular spaces. Similarly, Li et al. [15] conducted a detailed analysis of the hydrogen diffusion process during leakage from a mobile refueling station, using numerical modeling to evaluate key safety parameters such as the minimum safe explosion distance. The authors also paid attention to the influence of protective structures, including barrier walls, on the direction and intensity of hydrogen flow, using simulations to extrapolate fire hazard zones [16,17,18,19]. The correlation developed by Li [15] corresponds to the relationship between the high-dimensional spatial distribution of hydrogen concentration and low-dimensional variables such as leak rate, wind direction, and wind speed to predict hydrogen concentration in individual refueling stations. Despite the obtained results, the model did not provide effective prediction in the temporal dimension. Previous studies have mainly focused on achieving single goals, such as ensuring adequate model accuracy, but they were not able to capture the complex spatial–temporal dynamics of the phenomenon. In order to correctly represent the real operating conditions, it is necessary to consider both spatial and temporal variability in the analysis of hydrogen dispersion. The complexity of the hydrogen escape process and interactions with atmospheric conditions require the use of advanced computational fluid dynamics (CFD) methods. Cheng et al. [16] used a graph neural network (GNN) with a time-series plot to predict the evolution of hydrogen concentration based on data from a distributed network of sensors. Despite the novel approach, the model showed discrepancies in the generated two-dimensional concentration maps, which limited its precision in practical applications. Xiao et al. [17] used an artificial neural network (ANN) to estimate the vertical and horizontal diffusion ranges of a hydrogen plume, introducing features such as wind speed, leak intensity, and ground temperature into the model. Although the proposed approach allowed the estimation of hazardous hydrogen zones, the model lacked the ability to visualize the spatial distribution of concentrations in an intuitive and continuous manner. Moreover, the applied methods did not consider nonlinear interactions between environmental variables and the dynamic evolution of the cloud over time. To improve the prediction performance in the context of safety, future research should aim to integrate deep learning models with physical CFD models, which will allow them to achieve both high accuracy and consistency with real gas transport and diffusion processes. Considering time–space variability and real-time boundary conditions is crucial for the predictive monitoring of hydrogen hazards, which is enabled by CFD techniques. Meng and Khonsari [18] investigated the influence of surface texturing, specifically dimpled patterns, on the lubrication performance under hydrodynamic conditions. Their study focused on how dimples enhance the viscosity wedge effect by generating localized pressure build-up, which improves load-carrying capacity and reduces friction. Additionally, the authors incorporated the cavitation effect into their numerical model to more accurately simulate real lubrication scenarios, where vapor cavities can form within the lubricant film. The results demonstrate that optimized dimple geometry can significantly improve tribological performance by balancing pressure distribution and mitigating adverse cavitation impacts.

In one study [19], Chilou Zhou and Jinyang Zheng developed an FEM model to evaluate the impact of hydrogen-induced swelling on the sealing performance of rubber O-rings in high-pressure hydrogen storage systems. Their analysis demonstrated that increasing hydrogen pressure leads to enhanced swelling, which raises both contact stress, improving sealing capacity, and von Mises stress, potentially accelerating the mechanical degradation of the O-ring. The swelling effect was found to mimic an increase in compression ratio, highlighting the need to carefully consider swelling when determining optimal pre-compression to avoid seal failure due to cracking or relaxation. Additionally, the authors investigated the role of wedge–ring geometry, showing that a 45° wedge angle offers the best balance between preventing extrusion and enhancing sealing efficiency under elevated hydrogen pressures. Yamabe et al. (2009) analyzed the sealing performance of rubber O-rings in high-pressure hydrogen environments [20]. Their study highlights challenges such as hydrogen permeation, swelling, and leakage risks due to hydrogen’s small molecular size. The study emphasized the need for proper material selection and design to ensure seal integrity in hydrogen applications.

There is a lack in the literature of conditions that can accurately reflect the actual transition flow characteristics for high-pressure hydrogen flow in valves in the FSI approach. This gap is particularly critical, as hydrogen’s low molecular weight and high compressibility introduce complex flow behavior, especially in the transitional regime in turbulent states. Traditional models often rely on assumptions derived from air or inert gases, which may not be valid for hydrogen under high-pressure conditions. Experimental studies alone are not sufficient to fully represent the complex relationship between seal thickness and the acting hydrogen pressure stress in the contact zone of the quick-connect coupling. These phenomena are characterized by strong material and geometric nonlinearity, which requires advanced numerical models that take into account mechanical–thermal coupling. Only the integration of experimental data with FEM simulations allows an accurate representation of stress and deformation distributions, crucial for ensuring long-term leakage and safety in hydrogen systems. In the literature, there is a lack of comprehensive boundary conditions that are capable of accurately representing the transient flow characteristics of high-pressure hydrogen through valve systems. Existing models often rely on simplified assumptions that fail to capture the complex, unsteady behavior inherent to real hydrogen refueling scenarios. This gap limits the predictive accuracy of numerical simulations, particularly under dynamic loading and fast transients. Therefore, there is a critical need for experimentally validated, high-fidelity models tailored to the unique thermophysical properties of hydrogen at elevated pressures.

In this work, simulated high-pressure hydrogen flow in a quick connector with different operating conditions during transient states was simulated using Newtonian fluid assumptions and a turbulent flow model. Regions with polymer seals were fully connected using the fluid–structure interaction model. Taking into account rigid and elastic walls, contact stresses are analyzed using a thermal–mechanical FEM and FVM transient two-way solver. This approach enables the integration of flow field solutions obtained in Fluent with the static and dynamic structural analyses performed in ANSYS Mechanical 15.0. A transient two-way FSI simulation allows for a detailed study of the interaction between the fluid and the structure in conditions where both of these elements interact with each other. The numerical modeling presented in this study is based on experimental data obtained from tests conducted by CEJN on hydrogen quick couplings [21]. These experimental results provide critical validation benchmarks for assessing the performance and reliability of the simulated models. By integrating empirical findings, the simulations accurately reflect the real-world behavior of hydrogen quick-coupling systems under various operational conditions. This approach ensures that the numerical analysis remains aligned with industry-tested standards and enhances the predictive capabilities of the developed models.

## 2. Materials and Methods

In this paper, a multiphysics CFD model of a high-pressure hydrogen quick connector with an FFKM (perfluoroelastomer) O-ring seal is developed to investigate the thermal–mechanical stresses on the seal behavior in both steady-state and transient conditions of high-pressure hydrogen flow. The high-pressure quick coupler used in this study was sourced from CEJN AB (Skövde, Sweden), a manufacturer specializing in ultra high pressure connection technologies. According to technical documentation provided by the manufacturer, the coupler is designed for hydrogen gas applications up to 100 MPa, with features including non-drip disconnection, stainless steel construction, and integrated safety locking systems compliant with ISO standards [22]. The continuity equation for compressible fluid is

(1) $$\frac{\partial \rho }{\partial t}+\nabla \cdot \rho \bar{v}=0$$

where ρ—density, v—velocity, ∇—Nabla operator.

The momentum equation is

(2) $$\nabla \cdot \left(\rho \bar{v}\bar{v}\right)=-\nabla P+\nabla \cdot \mu \left[\nabla \bar{v}-\frac{2}{3}\nabla \cdot \bar{v}I\right]$$

where μ—dynamic viscosity, ∇—Nabla operator, I—unit tensor.

The energy equation is

(3) $$\nabla \cdot V(\rho E+P)=\nabla \cdot \left[\left(k_{T}+\frac{C_{p}\mu_{t}}{{\mathrm{Pr}}_{t}}\right)\nabla T+\mu_{eff}\left[\nabla \cdot \bar{v}-\frac{2}{3}\nabla \cdot \bar{v}\;\cdot I\right]\cdot \bar{v}\right]$$

where E—energy, $C_{p}$—specific heat capacity, $k_{t}—thermal\;conductivity$, $\mu_{eff}$—effective dynamic viscosity, ${Pr}_{t}$—Prandtl number, $\mu_{t}$—turbulent viscosity.

In the case of describing the gas phase, the volume-averaged Navier–Stokes equations are used. The Peng–Robinson equation of state is used to calculate the density of the gas phase:

(4) $$P=\frac{RT}{V_{m}-b}-\frac{a\alpha }{V_{m}^{2}+2bV_{m}-b^{2}}$$

where *a*, *b*—parameters that are calculated based on the critical data (critical temperature and pressure) and the acentric coefficient (*ω*), α—coefficients that depend on the type of gas and temperature, *V_m_*—molar volume.

The transient state of heat transfer in the fluid in one dimension for energy balance in a fluid volume element is

(5) $$\int_{V}\rho_{f}\frac{\partial h}{\partial t}dV+V\int_{A_{t}}G\frac{\partial h}{\partial z}dV=\int_{A_{t}}qA_{t}+\int_{V}\frac{G}{\rho_{f}}\left(\frac{\partial P}{\partial z}+f\frac{G}{2D_{h}\rho_{f}}\right)dV$$

where $\rho_{f}$—fluid density of element, h—specific enthalpy, G—mass flux, P—pressure, *f*—friction coefficient.

Seal surface contact pressure, taking account asperities, can be estimated by (6):

(6) $$p_{c}=\frac{4}{3}\eta E^{\prime }R^{1/2}\int_{h}^{\infty }\frac{1}{\sqrt{2\pi \sigma }}e^{-z^{2}/2\sigma^{2}}{\left(z-h\right)}^{3/2}dz$$

where η, R—the density and radius of asperities on the seal surface, respectively, $E^{\prime }$—equivalent elastic modulus.

### 2.1. Thermal–Mechanical Analysis

For a polymer O-ring seal, friction heat is generated during the movement of the valve spool and O-ring seal. Heat is transferred through the seal and valve spool by conduction, while convection governs heat exchange with the surrounding hydrogen gas. A 3-D energy equation is applied by taking account of the roughness effect to calculate the boundary layer of the contact between the gasket and the metal spool:

(7) $$\nabla \cdot (kh\nabla T_{m})-\rho chu_{m}\cdot \nabla T_{m}+\phi_{m}h+f_{c}p_{c}\bar{U}-q_{s}-q_{R}=0$$

where *c*—specific heat, *f_c_*—dry friction coefficient, $q_{s},\;q_{R}$—local heat flow into the seal and metal spool, k—thermal conductivity coefficient, $\bar{U}$—velocity vector, $\phi_{m}$—mass source.

### 2.2. Geometry

**Figure 1:**
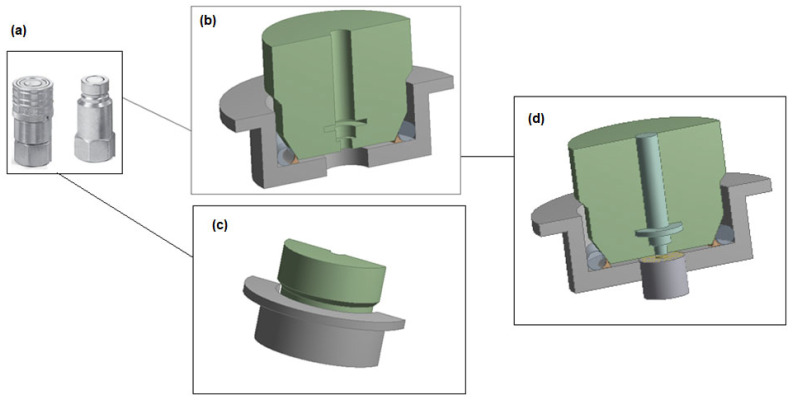
The diagram shows the geometry of the hydrogen quick coupler: (**a**) physical model; (**b**) cross-sectional view; (**c**) rear view; (**d**) view with hydrogen flow channel coupling structural–flow interface.

A quick-connect high-pressure hydrogen coupling made of stainless steel (AISI 316) is presented in Figure 1. The valve spool is shaped like a truncated cone and features a sealing O-ring positioned on its conical surface. During the manufacture of high-pressure couplings, surface hardening is applied to improve durability.

### 2.3. Meshing

A rigorous mesh independence study was conducted for the two-way fluid–structure interaction (FSI) simulation, which coupled a finite volume method (FVM)-based CFD solver for the fluid solver for the mechanical structure interaction. The fluid domain was discretized with meshes ranging from approximately 0.15 million to 2.3 million total elements, while the structural domain used about 150,841 to 2,290,852 finite elements to assess the impact of mesh resolution. A mesh independence study was carried out to verify that the obtained numerical results do not depend on the resolution used in the simulations. A mesh of 1,850,258 elements was enough to ensure the independence of model calculations. Mesh refinement was found to improve solution stability, as finer discretizations reduced numerical oscillations and enhanced the robustness of the strongly coupled solver. The grid resolution is presented in Table 1. Additionally, finer meshes enhanced the fidelity of the fluid–structure interface interactions by better resolving boundary-layer flows and stress gradients, leading to more accurate force and displacement transfer across the interface. To accommodate the moving structure, the CFD solver employed a dynamic mesh approach that allowed fluid mesh deformation at each coupling step in response to the structural displacements. This mesh motion was handled through a combination of O-ring-based smoothing and local remeshing techniques, which helped to maintain mesh quality and a conformal fluid–structure interface throughout the simulation. Nevertheless, ensuring high mesh quality during the strong coupling iterations was challenging, as large structural deformations could still distort the fluid mesh, leading to skewed or even inverted elements and potential numerical instabilities. The optimal mesh resolution was selected after observing changes in key FSI metrics such as peak structural displacement and fluid pressure load. Thus, the final numerical results were essentially mesh-independent, with no significant changes observed upon further mesh refinement, confirming that the chosen mesh resolutions for both fluid and structure provided a reliable and accurate solution.

### 2.4. Initial and Bondary Conditions

The initial condition for the simulation assumed a quick-connect hydrogen field, with pressure set to 0.1–20 MPa throughout the fluid domain and zero initial velocity, representing prefill ambient conditions. The structural domain was initialized with zero displacement and stress, corresponding to an undeformed but preloaded configuration of the elastomeric O-ring FKM material in its housing groove. At the fluid inlet, a total pressure boundary condition of 5–70 MPa and temperature of 298–700 K were applied, representing high-pressure hydrogen injection into the coupling during the refueling process. The outlet was defined as a static pressure boundary at 0.1–10 MPa, allowing the free outflow of gas and establishing the driving pressure differential. All fluid-side walls, including those in contact with the O-ring, were modeled as nonlinear temperature and compressibility gas-flow hydrogen, including variable physical properties (density, viscosity, enthalpy) dependent on temperature, taking into account strong thermal gradients in a very short time (transient). The model takes into account the deformability of the structure (e.g., O-ring, housing) and thermal conductivity in the structure. On the structural side, frictional contact was defined between the O-ring and adjacent metal surfaces to simulate real sealing behavior under pressure-induced deformation. The fluid–structure interface dynamically transferred pressure loads from the CFD domain to the FEM solver, and structural displacements were passed back as boundary motion to the CFD mesh using an ALE (Arbitrary Lagrangian–Eulerian) dynamic mesh formulation. The permeability coefficient of hydrogen was 5.32 × 10^−12^–8.41 × 10^−8^ (mol/m·s·Pa), depending on applied pressure and temperature. The diffusivity coefficient of hydrogen is on the order of 2.58 × 10^−10^–4.71 × 10^−8^ m^2^/s. This means that hydrogen spreads quite quickly in the FKM structure, but the permeability is still relatively low because it also depends on the solubility coefficient. The solubility coefficient for hydrogen in FKM is on the order of 1.47 × 10^−3^–8.98 × 10^−2^ (mol/m^3^·Pa). The hydrogen solubility coefficient (S) depends mainly on the material type, temperature, microstructure (cross-linking), and chemical nature of the medium. It does not depend directly on pressure, but affects the concentration of dissolved hydrogen at a given pressure.

### 2.5. Numerical Simulation

A two-way fluid–structure interaction (FSI) simulation was performed to evaluate the sealing performance of a hydrogen quick-connect coupling equipped with an FKM elastomeric O-ring. The simulation was conducted using ANSYS Fluent for the fluid domain and ANSYS Mechanical for the structural domain, coupled via System Coupling in a transient co-simulation scheme. Hydrogen flow at 20 MPa and 298 K was modeled using the Peng–Robinson equation of state, capturing real gas behavior under supercritical conditions. The Presto pressure-based solver in Fluent was employed in combination with the PISO (Pressure-Implicit with Splitting of Operators) algorithm for pressure–velocity coupling, enabling the accurate transient resolution of compressible hydrogen flow. The SST k–ω turbulence model was applied with enhanced wall treatment, and the fluid mesh was refined to maintain y+ values between 1 and 5, ensuring the proper resolution of the viscous sublayer. Boundary conditions included a pressure inlet (5–20 MPa), pressure outlet (0.1–10 MPa), and adiabatic slip wall conditions. The O-ring’s structural response to gas loading was modeled using a Mooney–Rivlin hyperelastic material model, calibrated for FKM rubber behavior. Frictional contact between the O-ring and metallic groove was defined, while the metal components were treated as nonlinearly elastic. A transient time step of 0.001 s was used over a 10 s simulation, sufficient to capture dynamic sealing behavior under pressure loading. The fluid mesh employed inflation layers with prism elements near walls, while the structural mesh used second-order solid elements in the O-ring domain. The Pressure-Implicit with Splitting of Operators (PISO) algorithm was used for pressure–velocity coupling because it is better for unsteady flows for large accelerations. It uses two pressure corrections, which allows faster convergence without the need for relaxation. The computation time is longer, but it is more accurate, especially for unsteady and dynamic flow meshing, which models the moving interfaces of the seal with the valve elements. An ALE-based dynamic mesh was used in Fluent to accommodate deformations transferred from the structural domain at each coupling iteration. Convergence within each time step was enforced through Fluent residuals below 10^−3^, structural displacement convergence below 10^−6^ m, and a coupling interface tolerance of 10^−3^. Typically, 3–5 FSI iterations per time step were required to achieve full convergence. The combination of the Presto solver and PISO algorithm provided high-fidelity pressure field prediction, essential for capturing localized loading on the sealing surface. To maintain numerical stability, the CFL (Courant–Friedrichs–Lewy) number was controlled to remain below 1 throughout the domain, particularly near the fluid–structure interface. A summary of the simulation parameters is shown in Table 2. This ensured that the transient flow features were properly resolved without violating stability limits in regions of high velocity gradient. This simulation framework enabled prediction of O-ring deformation, contact pressure, and potential leakage zones. The methodology supports the design optimization and safety verification of hydrogen quick-connect couplings under high-pressure operating conditions in accordance with current hydrogen component standards.

### 2.6. Fluid–Structure Interaction Coupling Method: FEM–FVM

This study involves two-way fluid–structure interaction (FSI), which is widely employed in the numerical analysis of high-pressure hydrogen quick-connect couplings, particularly in evaluating the mechanical response of elastomeric O-ring seals subjected to dynamic gas loading. For hydrogen flow at pressures around 5–20 MPa, the accurate modeling of coupled interactions between the gas domain and structural deformation is essential. The Finite Volume Method (FVM) is utilized in ANSYS Fluent to resolve transient compressible hydrogen flow, incorporating a real-gas equation of state, such as Peng–Robinson, which captures the thermodynamic behavior under supercritical conditions. The mechanical response of the O-ring is simulated using the Finite Element Method (FEM) in ANSYS Mechanical, employing hyperelastic material models (Mooney–Rivlin) to represent FKM-based fluoroelastomers with hydrogen compatibility. The coupling interface between the fluid and solid domains is defined within ANSYS Workbench using System Coupling, which facilitates iterative data exchange between the solvers. In each time step, pressure and shear forces computed by Fluent are transferred to the structural domain, inducing local deformations of the O-ring, typically in the range of 0.2–0.5 mm. These deformations are then communicated back to Fluent as mesh displacements using the ALE (Arbitrary Lagrangian–Eulerian) formulation, dynamically updating the fluid domain geometry. This process is repeated within each time step until convergence criteria are satisfied, following a tight coupling algorithm. For transient simulations, a typical tanking event duration of 10 s is modeled with a time step of 0.01 s, capturing the evolution of pressure, velocity, and structural response. The initial system temperature is set at 298 K, though thermal effects from hydrogen expansion (e.g., the Joule–Thomson effect) may lead to temperature drops that influence seal performance. The geometry of the coupling includes a flow passage and a retention groove for the O-ring in a “gland” configuration. Validation focuses on contact pressure distributions, structural displacements, and potential leakage zones arising from an excessive deformation or loss of sealing integrity. The hydrogen flow is treated as compressible, unsteady, and turbulent, with turbulence resolved using the k–ω SST model to accurately capture near-wall behavior. Structural contact analyses are incorporated to evaluate the seal interface under varying pressure loads, including contact separation or partial sealing scenarios. In two-way FSI simulations involving a hydrogen quick-connect coupling with an elastomeric O-ring, dynamic mesh techniques are essential for accurately capturing the fluid-domain deformation caused by the structural response of the seal. The dynamic mesh in ANSYS Fluent allows the fluid mesh to adapt in real time to the displacements received from the structural solver (ANSYS Mechanical). This is especially critical in areas near the fluid–structure interface, where mesh distortion can compromise solution accuracy and stability. The mesh motion is typically handled using spring-based smoothing, local remeshing, or layering, depending on the magnitude and nature of the deformation. For simulations involving large displacements of the O-ring (e.g., 0.3–0.5 mm), remeshing is often required to maintain element quality. Dynamic mesh is also essential when using the ALE (Arbitrary Lagrangian–Eulerian) formulation, enabling the fluid mesh to follow the moving boundaries imposed by structural deformation. Proper dynamic mesh control ensures numerical convergence and the reliability of two-way FSI results, especially under transient high-pressure hydrogen loading conditions. The simulation results support the optimization of coupling geometry, O-ring material selection, and operational pressure limits to mitigate failure modes such as hydrogen permeation, extrusion, or fatigue-induced damage. The methodology is applicable for verifying compliance with international hydrogen component standards, following the guidelines specified in ISO 19880-3 [22], which govern the design of high-pressure hydrogen systems. In addition, hydrogen permeation and the long-term degradation of elastomeric seals can be evaluated by extending the model to include microleakage and material fatigue behavior. Thermal, chemical, and frictional effects can be integrated into the model depending on the level of complexity required. Mesh quality is critical, with typical meshes involving 758,458 elements for CFD and 366,147 elements for FEM, ensuring resolution at the fluid–solid interface. Ultimately, two-way FSI simulation enables a predictive evaluation of sealing performance and structural integrity in hydrogen quick-connect couplings, enhancing the safety, durability, and reliability of high-pressure hydrogen refueling systems.

## 3. Results and Discussions

**Figure 2:**
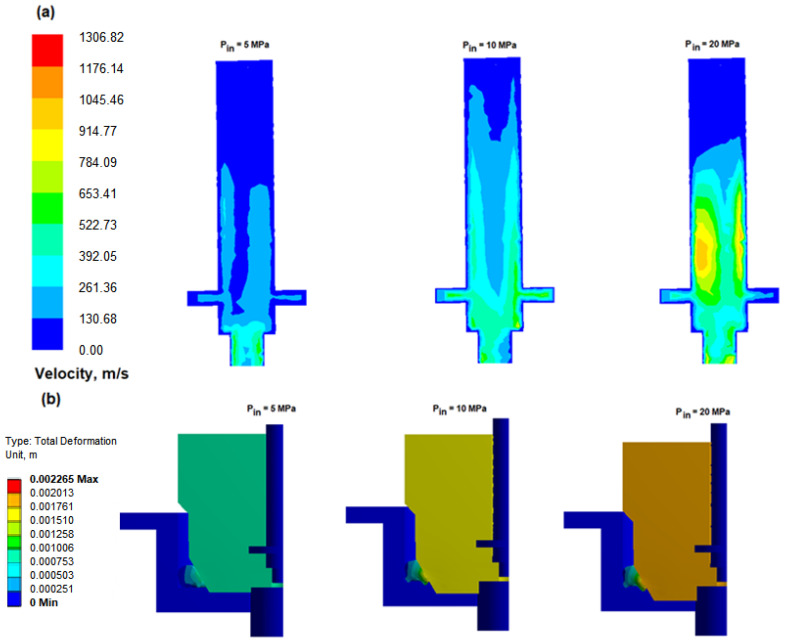
Velocity distribution in the hydrogen quick-connector channel: (**a**) influence of different inlet pressures; (**b**) corresponding deformations of the polymer O-ring seal (mm).

Figure 2 shows the distribution of hydrogen flow rates in the hydrogen quick-connector channel for different inlet pressures: 5, 10 and 20 MPa, along with the corresponding deformation shapes of the polymer O-ring seal (in mm). As the hydrogen inlet pressure increases, increasing seal deformations are observed. At high flow rates, hydrogen exerts greater pressure on the O-ring surface, which leads to its significant deformation. Due to the low viscosity and density of hydrogen, the Reynolds number increases very quickly at high flow rates and small channel diameters, which favors the transition of the flow into a turbulent state. Additionally, hydrogen as a gas with very low viscosity easily separates from the surface (the phenomenon of stream separation) with a high degree of hydrogen compressibility, which favors the formation of unstable rotating structures. The use of rounded edges and gradual changes in the channel diameter can effectively reduce the phenomenon of stream separation and improve flow stability. The relationship between the hydrogen flow rate and pressure causes the nonlinear seal deformation. For a pressure of 10 MPa, the total deformation of the seal is 1.51 mm, while, at 20 MPa, it increases to 2.2 mm. More compression leads to excessive deformation and accelerated wear, while too little compression leads to system connection leaks.

**Figure 3:**
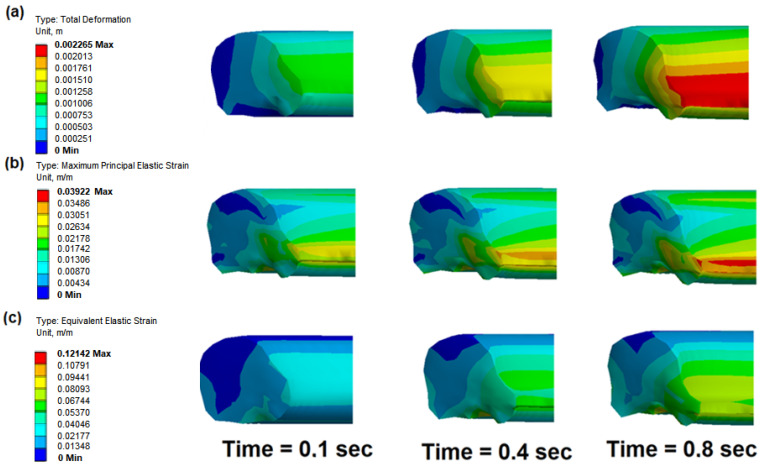
Effect of different transient states on the gasket temperature distribution during hydrogen flow: (**a**) total deformation of the polymer seal; (**b**) maximum principal elastic strain of polymer gasket; (**c**) equivalent elastic strain of polymer gasket (operating conditions: P = 15 MPa, temperature = 43 °C).

Figure 3a presents the total deformation of a polymer seal under unsteady-state conditions during hydrogen flow through a high-pressure quick connector. Within 0.4 s, high-pressure hydrogen reaches the valve spool surface, resulting in a rapid increase in both flow rate and pressure. These dynamic conditions significantly influence the deformation behavior of the polymer seal, as governed by the inlet pressure. Figure 3b shows the maximum principal elastic strain, which is a key parameter in evaluating material response under mechanical loading. This metric is particularly relevant in the design of polymeric seals such as thermoplastic elastomers (TPEs), which are exposed to pressure, temperature, contact forces, and cyclic deformation. This provides insight into whether the material remains within its elastic regime or approaches the onset of plastic deformation or structural failure. For TPE materials, elastic deformation typically ranges between 5 and 10%; exceeding this range may lead to permanent deformation. Finite Element Method (FEM) analysis reveals that, at 0.8 s, the maximum principal elastic strain reaches 0.03922 m/m. This value highlights the most critical zones of local strain concentration. Additionally, the equivalent elastic strain offers an energy-based evaluation of elastic deformation, accounting for all directional components. It serves as a comprehensive indicator of material safety and helps to assess whether the seal operates within allowable limits. Figure 3c shows the equivalent elastic strain distribution, interpreted in this context as a scalar measure of total elastic deformation that incorporates strain components in all directions. The peak equivalent elastic strain at 0.8 s reaches approximately 0.1214 m/m, which falls well within the expected range for FFKM seals under high-pressure, high-temperature hydrogen exposure. As the Mooney–Rivlin hyperelastic model was employed, assuming fully reversible elastic behavior, the equivalent strain values are used here solely to characterize the deformation intensity, without suggesting energy accumulation or irreversible damage. This approach is appropriate for perfluoroelastomers, which are specifically engineered to resist chemical degradation and exhibit minimal stress relaxation and compression set under demanding service conditions. In O-ring seals, neglecting minimum principal strain could overlook areas where the material is overstressed in compression, leading to long-term degradation mechanisms like compression set or structural instability. Therefore, analyzing both maximum and minimum principal strains provides a comprehensive view of the stress–strain state, ensuring that both tensile-related failures and compressive damage risks are properly assessed in complex loading scenarios, such as those present in fluid–structure interaction (FSI) with high-pressure hydrogen environments.

**Figure 4:**
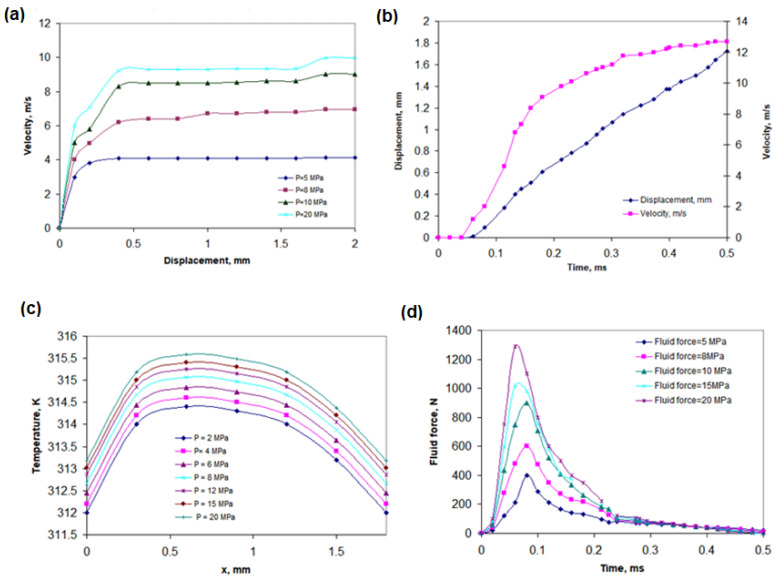
The influence of individual operating parameters on the seal behavior: (**a**) displacement of gasket, depending on the incoming hydrogen pressure; (**b**) relationship between gasket displacement and velocity during hydrogen flow in unsteady state; (**c**) relationship between temperature and gasket displacement as a function of the incoming hydrogen pressure; (**d**) relationship between fluid force and time during quick connecting.

Figure 4a presents the displacement of the gasket as a function of incoming hydrogen pressure. As the inlet pressure of hydrogen increases, both its velocity and the degree of gasket displacement rise. At 5 MPa, the displacement reaches 0.31 mm, while, at 20 MPa, it increases to 0.49 mm. This behavior results from the direct mechanical interaction between the gas flow and the polymer seal. As pressure increases, the force exerted on the gasket intensifies, leading to greater deformation. This deformation directly influences sealing performance, particularly under unsteady flow conditions, where rapid pressure variations generate dynamic stresses on the O-ring. Figure 4b illustrates the relationship between gasket displacement and flow velocity during the initial phase of unsteady hydrogen flow. The displacement increases over time, correlating with the rise in hydrogen velocity. Figure 4c shows the relationship between temperature and gasket displacement as a function of incoming hydrogen pressure. In a very short time, intense molecular friction and gas compressibility lead to local gas compression. This results in the formation of shockwaves and rapid hydrogen flow through the sealing gap. Figure 4d depicts the relationship between fluid force and time during the quick connection process for different inlet pressures. At an inlet pressure of 20 MPa, the fluid force can reach up to 1300 N.

**Figure 5:**
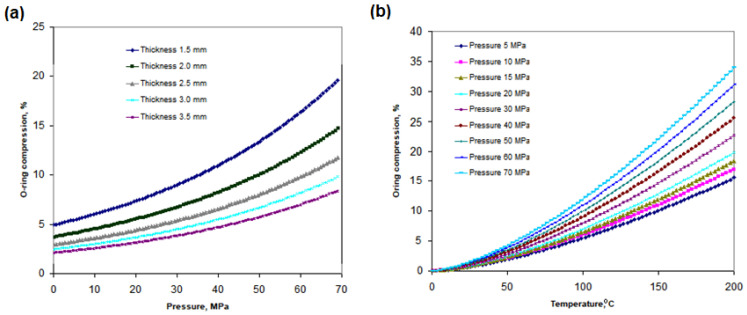
Effect of temperature and pressure on O-ring compression: (**a**) compression of the O-ring, depending on the pressure of the flowing hydrogen and the O-ring thickness; (**b**) compression of the O-ring, depending on the flowing hydrogen pressure and temperature.

Figure 5a shows the compression of the O-ring as a function of hydrogen pressure and O-ring thickness. As the thickness of the O-ring increases, its percentage compression decreases. A thicker O-ring exhibits greater stiffness, making it more resistant to compression within the sealing gap. However, this reduced compression may lead to a smaller effective contact area, which can negatively impact the sealing performance under high hydrogen pressure. High gas pressure (in this case, hydrogen) pushes the O-ring against the sealing surfaces, increasing its compression and improving tightness. At 40 MPa, the compression for a 3.5 mm thick O-ring is 4.23%, while, for a 2 mm thick O-ring, under the same pressure, it reaches 7.23%. Figure 5b illustrates how O-ring compression changes, depending on the applied pressure and temperature. With increasing pressure and temperature, the compression of the O-ring also increases. At 70 MPa, the compression is 18.39% higher than at 5 MPa, indicating a strong correlation between thermal and pressure loading and the sealing behavior of the O-ring gasket.

**Figure 6:**
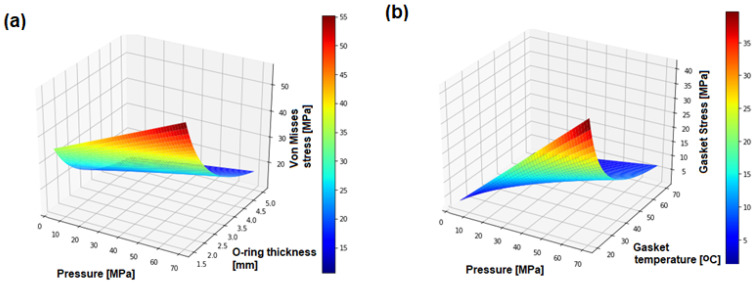

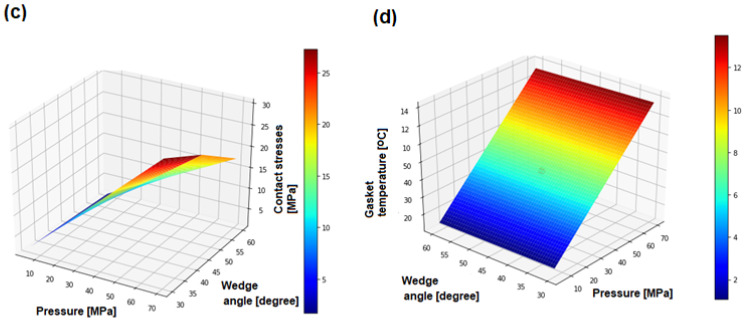
Parametric study on the thermomechanical response of FFKM seals under high-pressure hydrogen flow: (**a**) effect of hydrogen pressure and O-ring thickness on von Mises stress in a polymer seal; (**b**) effect of hydrogen pressure and seal temperature on seal stress; (**c**) effect of hydrogen pressure and wedge angle on contact stress in a polymer seal; (**d**) effect of hydrogen pressure and wedge angle on O-ring gasket temperature.

Figure 6a shows the influence of hydrogen pressure and O-ring thickness on von Mises stress in the polymer seal. In hydrogen quick connectors, the O-ring is subjected to increasing pressure, which directly affects its level of compression. As the pressure rises, the stress within the seal increases. However, with increasing O-ring thickness, these stresses tend to decrease. Thicker seals exhibit greater stiffness, making them more resistant to compression. Nevertheless, they may conform less effectively to surface irregularities, which can negatively affect sealing performance. For instance, at 60 MPa and with a 5 mm thick O-ring, von Mises stress remains below 20 MPa, indicating improved load distribution. Figure 6b illustrates the influence of seal temperature and inlet hydrogen pressure on stress distribution within the seal. Figure 6b presents the distribution of von Mises equivalent stress in the FFKM polymer seal as a function of hydrogen pressure and gasket temperature. This approach enables the evaluation of whether the stress levels remain within the safe elastic limits of the FFKM material, without implying material yielding or irreversible deformation. The observed decrease in equivalent stress with increasing temperature is primarily attributed to the reduction in the elastic modulus of FFKM at elevated temperatures, resulting in greater material compliance under load. As the temperature increases, the internal stress in the polymer seal decreases. This is primarily due to the reduction in the elastic modulus of the material, making it softer and more compliant, thus requiring less force to deform. In addition, a stress relaxation phenomenon occurs, in which the internal stress gradually decreases under constant strain. This process is accelerated by higher temperatures. Although elevated temperature can reduce internal stress and improve flexibility, it should be noted that excessive heating may cause irreversible damage, such as material degradation or a decline in sealing performance. Figure 6c presents the effect of hydrogen pressure and wedge angle on contact stress. Smaller wedge angles (e.g., 30°) generate higher contact stresses, while larger angles (e.g., greater than 50°) result in lower stresses. While higher contact stress can improve sealing effectiveness at elevated pressures, it may also lead to faster seal wear. In contrast, lower contact stress improves durability but can compromise sealing integrity. Therefore, selecting an appropriate wedge angle is essential to balance longevity and performance. The optimal angle for hydrogen quick connectors is approximately 48°. At 30°, contact stress is the highest, as the normal force on the contact surface is greatest. At 45°, a compromise is achieved between sealing force and contact area. At 60°, contact stress is the lowest, due to the reduced normal component of the force, which minimizes local pressure on the seal. In spite of the benefits of lower contact stress at larger angles, this configuration may be unsuitable for high-pressure environments. For such conditions (e.g., 70 MPa), smaller wedge angles (30–45°) are recommended to increase contact pressure and ensure leak-tightness. By contrast, at lower pressures (<30 MPa), larger wedge angles (e.g., 60°) may be advantageous to reduce mechanical loading and prolong seal life. Figure 6d illustrates the temperature increase in the polymer seal as a function of wedge angle and hydrogen pressure. At 45 MPa and a wedge angle of 45°, the seal temperature remains within an optimal range. Nonetheless, connector geometry and exposure time must also be considered. At high pressures, hydrogen molecules exert significant force on the seal, leading to localized heating. Moreover, frictional interactions at the contact interface may generate additional heat. As a result, the careful selection of the seal material and wedge geometry is crucial to ensure durability and performance under demanding hydrogen service conditions.

## 4. Conclusions

In this paper, a multiphysics CFD model of a high-pressure hydrogen quick connector using an O-ring seal made of FFKM (perfluoroelastomer) was developed. The aim of the work was to investigate the thermal–mechanical stress distribution and seal behavior in both steady and unsteady states. The O-ring seal model, analyzed for thermal–elastic properties in the sealing zone, was developed using the Finite Element Method (FEM) coupled with the Finite Volume Method (FVM) applied to model the hydrogen flow under pressure. The Reynolds equation, energy equation, and heat conduction equation were solved using the weighted residuals method in the finite element approach. A parametric analysis was performed to determine the effect of operating conditions on the thermal effects and thermo-elastohydrodynamic properties of O-ring seals in a hydrogen quick connector. It has been shown that the thermal effect has a significant impact on the temperature distribution in the seal and the viscosity of hydrogen at high pressure in the sealing zone. The maximum zone of temperature increase is located near the sealing surface, where heat conduction dominates, while the convection effect prevails in the zone of relatively constant temperature, located closer to the sealing wedge surface. In addition, under conditions of rapid pressure jumps, the seal shows an increased risk of failure due to extreme contact stresses. It is emphasized that these conditions must be taken into account in the design of seals for hydrogen quick connectors. It should also be noted that the steady state was taken into account in the current model, but, in real applications, the pressure in the system changes dynamically over time.

Main conclusions from the work are as follows:

1. The hydrogen pressure at the inlet has a significant impact on the operation of the valve spool during opening. The increase in pressure causes an increase in the force acting on the spool and, thus, the acceleration, which shortens the time needed to reach the end position;
2. Simulations have shown that increasing the hydrogen inlet pressure from 5 MPa to 20 MPa results in a significant increase in O-ring deformation. The maximum deformation reached 0.02265 mm, which significantly affects the tightness and durability of the system. The effect of hydrogen pressure on O-ring stresses is nonlinear—for a thickness of 5 mm, von Mises stresses did not exceed 20 MPa, while; for thinner seals; they increased significantly with increasing pressure;
3. It was observed that the hydrogen flow velocity can exceed 1300 m/s, which favors the formation of turbulence and unstable rotating structures in the quick-release space;
4. Within 0.08 ms, hydrogen at a pressure of 18 MPa completely filled the chamber before the sealing zone. The pressure on the spool surface reached a maximum value of 12 MPa, then dropped rapidly to 5.6 MPa (at 0.18 ms), which confirms the dynamic and cyclic nature of the loads;
5. The increase in temperature causes a decrease in the stresses in the seal, which results from the reduction of the material’s modulus of elasticity and the relaxation effect. For a temperature of 23 °C and a pressure of 15 MPa, the von Mises stresses did not exceed 20 MPa;
6. The analysis of the effect of the O-ring thickness showed that, at a pressure of 40 MPa, the compression for a thickness of 3.5 mm was 4.23%, while, for 2 mm, it was as much as 7.23%. This shows the importance of the selection of the seal geometry in high-pressure applications;
7. The wedge angle has a large effect on the value of the contact stress. For an angle of 30°, the stress reaches a maximum level, which ensures good tightness, but increases wear. The optimum wedge angle was determined to be 48°, as a compromise between sealing efficiency and durability;
8. Fluid forces at high hydrogen flow (up to 20 MPa) cause a valve spool displacement of up to 0.30 mm, which significantly affects the pressure distribution and flow velocity over time. At pressures greater than 60 MPa, adiabatic compression can occur, causing a local temperature increase in the sealing zone. Such a temperature increase can lead to the degradation of the elastomer material in the long term;
9. The use of appropriately selected design parameters such as channel geometry, rounded transitions, orifice length, and O-ring material allows for the reduction of stream separation and turbulence phenomena, which significantly increases the reliability and durability of quick connectors in hydrogen applications.

To mitigate the adverse effects of pressure surges and excessive wear, it is recommended to implement adaptive pressure control mechanisms or pressure relief systems specifically designed for high-pressure hydrogen environments. Such systems would enhance the safety and durability of quick couplers, especially in hydrogen refueling stations where sudden pressure changes are frequent. Additionally, optimizing O-ring material properties is essential for maintaining durability under cyclic loading. Future research should focus on developing composite or hybrid elastomer materials that exhibit stable mechanical properties under varying pressure and temperature conditions. Incorporating adaptive sealing compounds with temperature compensation capabilities would significantly improve sealing performance, reducing the risk of failure during temperature fluctuations.

## Figures and Tables

**Table 1:** Resolutions for grid independence check.

Grid Number	Cell Size (mm)	Total Elements (mln)
1.	0.43117	0.150841
2.	0.12847	0.784652
3.	0.10877	1.118012
4.	0.09652	1.274115
5.	0.04258	1.352869
6.	0.01182	1.544933
7.	0.00852	1.850258
8.	0.001774	2.290852

**Table 2:** Main summary of CFD simulation parameters.

Parameter	Value/Name
Time stepping	Courant number-dependent
Courant number	0.48
Time solver	Euler–Lagrange
Pressure–velocity coupling scheme	PISO
Gas equation of state	Peng–Robinson
Kinematic viscosities	Temperature dependency
Transient time step	0.001 s

## Data Availability

The data presented in this study are not publicly available due to confidentiality.
